# Towards a Better Understanding of Texturization during High-Moisture Extrusion (HME)—Part I: Modeling the Texturability of Plant-Based Proteins

**DOI:** 10.3390/foods12101955

**Published:** 2023-05-11

**Authors:** Elisabeth Högg, Cornelia Rauh

**Affiliations:** Department of Food Biotechnology and Food Process Engineering, Technische Universität Berlin (TU Berlin), 14195 Berlin, Germany

**Keywords:** plant-based proteins, high-moisture extrusion, HMMA, thermophysical properties, texturization indicator, texturability

## Abstract

This study focused on predicting high-moisture texturization of plant-based proteins (soy protein concentrate (SPC), soy protein isolate (SPI), pea protein isolate (PPI)) at different water contents (57.5%, 60%, 65%, 70%, and 72.5% (*w*/*w* db)) to optimize and guarantee the production of high-moisture meat analogs (HMMA). Therefore, high-moisture extrusion (HME) experiments were performed, and the texture of the obtained high-moisture extruded samples (HMES) was sensory evaluated and categorized into poorly-textured, textured, or well-textured. In parallel, data on heat capacity (c_p_) and phase transition behavior of the plant-based proteins were determined using differential scanning calorimetry (DSC). Based on the DSC data, a model for predicting c_p_ of hydrated, but not extruded, plant-based proteins was developed. Furthermore, based on the aforementioned model for predicting cp and DSC data on phase transition behavior of the plant-based proteins in combination with conducted HME trials and the mentioned model for predicting cp, a texturization indicator was developed, which could be used to calculate the minimum threshold temperature required to texturize plant-based proteins during HME. The outcome of this study could help to minimize the resources of expensive extrusion trials in the industry to produce HMMA with defined textures.

## 1. Introduction

The current trend to produce sustainable high-moisture meat analogs (HMMA) with high-moisture extrusion (HME) is pushing scientific research to better understand and evaluate the texturization process in the extruder barrel (=screw section) as well as in the cooling die (=cooling die section) to develop meat analog products with defined textures [1,2,3,4].

Based on the literature and our definition, the following terminology should be used to describe and define HME products: only products with a defined texture that closely resembles meat products being mimicked should be referred to as HMMA. All other samples should generally be referred to as high-moisture extruded samples (HMES), HME samples, and extrudates or texturates [5,6,7,8,9]. However, the aforementioned terms, such as HMES, HME samples, extrudates, or texturates, can include HMMA, as mentioned above.

As thermodynamic approaches, like differential scanning calorimetry (DSC), can provide valuable information on food systems’ possible state and potential behavior [10], one approach to guarantee the production of HMMA is to study the importance and influence of thermophysical properties of plant-based protein materials and their HMES in combination with textural evaluation methods. Here, DSC analysis offers a great opportunity to monitor heat effects (e.g., heat capacity and phase transitions) in proteins. By using the aforementioned approaches, certain texturization domains for desired HMES textures might be calculated to achieve an optimized HME outcome.

The primary focus of this study was on the screw section of the HME process rather than on the cooling die, wherefore in this study, the term phase transition refers to a first-order phase transition with a change in the physical state from solid to liquid of the protein involved [11,12]. Therefore, in this study, it was investigated whether the enthalpy input required in the screw section of an extruder could be calculated to achieve protein melting and, thus, to produce HMES with defined multilayered, fibrous structures since protein melting represents the initial state for HME texturization [13,14]. Protein melting leads to swelling, dissolution, and unfolding of proteins, followed by polymerization, although depolymerization may occur as well, in which the proteins form intermolecular bonds, including both non-covalent and covalent bonds, like disulfide bonds [9,15,16,17,18,19]. The proteins are then stabilized due to cooling in the cooling die and create fibrous structures through forming more hydrogen bonding and hydrophobic and electrostatic interaction [17,19,20,21]. Due to the thermophysical effects during protein unfolding leading to a strong change in enthalpy at the transition point, in this study, it was hypothesized that the total energy a plant-based protein must experience in the extruder barrel should be greater than its melting enthalpy measured by DSC in order to achieve complete protein melting, and thus, defined texturization during HME.

To determine and calculate the thermophysical properties of plant-based proteins, a fundamental thermal characterization of soy protein concentrate ALPHA^®^ 8 IP was conducted. Thus, the DSC results were evaluated in terms of specific heat capacity, melting temperature, and melting enthalpy. The melting temperature ™ is a common descriptor to quantify the stability of proteins and is defined as the temperature at which the protein denatures (=loss of native form), which is accompanied by the unfolding of this protein [22,23,24]

The melting enthalpy (ΔH_m_) is defined as the amount of heat required for converting the native state of a protein to the unfolded state, meaning ΔH_m_ is the enthalpy needed for the unfolding reaction at T_m_ [25]. As the unfolding of a protein represents an endothermic process, the term ∆H_m_ will be positively charged. Melting po™(T_m_) and enthalpy of unfolding (ΔH_m_) are commonly used to characterize protein stability and protein denaturation, respectively [26].

Therefore, based on the obtained data from DSC measurements and in combination with HME experiments, data-driven prediction models were generated, and a texturization indicator was derived to predict the texturization conditions of plant-based protein materials prior to HME to produce HMMA with multilayered and fibrous structures. This approach was validated by HME experiments not only for soy protein concentrate ALPHA^®^ 8 IP but also for other proteins (soy protein concentrate PROCON^®^ 2000 IP, soy protein isolate Wilpro D150 as well as pea protein isolate Pisane^®^ C9).

Being able to predict texturization properties of plant-based protein materials prior to HME processing would minimize resources for research and development of meat analogs and would increase ingredient flexibility and process stability. To date, the screening of plant-based proteins relies heavily on empirical knowledge, and HME experiments are mainly based on the concept of trial and error [27,28].

To the best of our knowledge, no studies exist investigating prediction models for high-moisture texturization of plant-based proteins.

## 2. Material and Methods:

### 2.1. Materials

A soybean protein concentrate (SPC ALPHA^®^ 8 IP) was purchased from Solae Europe S.A. (Geneva, Switzerland). The food industry often uses this plant-based raw material as a benchmark reference ingredient for HME. Within this paper, the soy protein concentrate ALPHA^®^ 8 IP will be referred to as ‘SPC Alpha 8’. Further, for validation purposes of the derived prediction models, the soy protein concentrate PROCON^®^ 2000 IP (Solae Europe S.A., Geneva, Switzerland), the soy protein isolate Wilpro D150 (Yihai Kerry (Qinhuangdao) Protein Industries Co., Ltd., Qinhuangdao, China), as well as the pea protein isolate Pisane^®^ C9 (COSUCRA Groupe Warcoing S.A., Warcoing, Belgium) were used. Within this paper, the soy protein concentrate PROCON^®^ 2000 IP, the soy protein isolate Wilpro D150, and the pea protein isolate Pisane^®^ C9 will be referred to as ‘SPC Procon’, ‘SPI Wilcon’, and ‘PPI Pisane’, respectively. All analytical methods described in the following for SPC Alpha 8 were also conducted for the plant-based proteins used in the validation study. A detailed description of the experimental plan of the validation study can be found at the end of the Material and Method section. Tap water (Berlin, Germany) was used to adjust the samples to the target water contents.

### 2.2. Sample Preparation of SPC Alpha 8

The intrinsic moisture of the plant-based protein SPC Alpha 8 was considered and measured beforehand using a moisture analyzer (MA35, Sartorius AG, Goettingen, Germany). SPC Alpha 8 was mixed with tap water (*w*/*w*) to obtain the target water contents of 60%, 65%, and 70% (db). The water contents applied corresponded to the water contents used in the high-moisture extrusion experiments for texturization (Table 1). In those experiments, SPC Alpha 8 was directly mixed in the extruder barrel with the needed amount of tap water.

After mixing, the hydrated SPC Alpha 8 samples were stored in airtight containers at 4–8 °C for at least 18 h to ensure full hydration.

### 2.3. Determination of Specific Heat Capacity of SPC Alpha 8 by µDSC

The specific heat capacity was measured in the range of 40 to 115 °C with a µDSC (µDSC 7 EVO, SETARAM Instrumentation, Caluire, France) at constant pressure (dp=0). The cell was filled with a 0.6500 g (±0.0010 g) sample. The scanning rate was 0.2 °C min^−1^. A minimum of two calorimetric measurements was performed. All data were processed with CALISTO-Software (Version v1.097, SETARAM Instrumentation, Caluire, France). To evaluate the accuracy of the microcalorimeter prior to sample analyses, the specific heat capacity c_p_ of water was determined and compared with the literature value. Further, a blank test was performed with an empty cell. All data were corrected with the uncertainty between the measured specific heat capacity c_p_ of water and its literature-based specific heat capacity c_p_ [29,30,31].

To study the reversibility of the µDSC measurement and the influence of possible denaturation reactions on c_p_ of plant-based proteins, a preliminary study with SPC Alpha 8 moistened to 60% (*w*/*w* db) was conducted. Therefore, the following procedure was applied: (1) an initial µDSC scan was performed; (2) the sample was cooled to 40 °C within 1.5 h; (3) equilibrated at 40 °C for 1 h, and (4) rescanned [31]. The results of the initial run and its rerun showed no significant differences in heat capacity (*p* < 0.05).

### 2.4. Determination of Phase Transition of SPC Alpha 8 by DSC

To study the phase transition of hydrated SPC Alpha 8, differential scanning calorimetry (DSC) was performed using a 204 F1 Phoenix calorimeter fitted with an intracooler (NETZSCH-Gerätebau GmbH, Selb, Germany). Ten mg (±1 mg) of the sample was weighed in an aluminum pan (NETZSCH-Gerätebau GmbH, Selb, Germany) and hermetically sealed. The samples were heated from 10 to 170 °C with a heating rate of 5 °C min^−1^. The obtained thermograms were evaluated by NETZSCH Proteus—Thermal Analysis—Version 7.1.0 software (NETZSCH-Gerätebau GmbH, Selb, Germany) in terms of onset temperature (T_onset_), end temperature (T_end_), melting temperature (T_m_), melting enthalpy (ΔH_m_), and the total applied enthalpy (ΔH_DSC_) during each DSC measurement. The experiments were performed at least in double determination, and arithmetic means, as well as the corresponding standard deviations, were calculated.

### 2.5. High-Moisture Texturization of SPC Alpha 8

For the HME experiments of SPC Alpha 8, a laboratory co-rotating twin screw extruder ZSK 25 from Werner & Pfleiderer (Coperion GmbH, Stuttgart, Germany) with an outer screw diameter of Da = 25 mm and a length–outer diameter ratio of L/Da = 28.8 was used.

A central composite design (CCD) matrix was applied in designing the experiments based on processing data available in the literature (Table 1). The extruder variables (1) screw speed (220–400 rpm), (2) water content (60–70%), (3) mass flow rate (6–13 kg/h), and (4) barrel temperature (120–160 °C) were set as independent variables of the CCD. The cooling die remained unchanged with a geometry of 250 × 60 × 9 mm^3^ (L × W × H) and a cooling temperature of 40 °C. Tap water was added into the second extruder barrel to adjust moisture content (*w*/*w* db). The comprehensive study simulated by the CCD using the Design-Expert software 10 (Stat-Ease, Minneapolis, MN, USA) incorporated 27 individual experiments listed in Table 1.

The dependent variables of the CCD were material temperature (T_in_), extruder pressure (p_in_), and specific mechanical energy (SME). Those variables are defined as system parameters of the extruder response [32] Material temperature (T_in_) and extruder pressure (p_in_) were measured at the extruder exit, directly before the inlet of the cooling die, using a thermocouple type J (Voltcraft, Wollerau, Switzerland) and a melt pressure sensor MDT422 (Dynisco Instruments, LLC, Franklin, MA, USA), respectively. *SME* was calculated according to Meuser and van Lengerich [32] via Equation (1):

(1) $$SME[Wh/kg]=\frac{\frac{n}{n_{max}}\times (\frac{M_{d}-M_{d,unload}}{100})}{\dot{m}}*P_{max}$$

where *n* is the actual screw speed in min^−1^, and *n_max_* is the maximum screw speed of 500 min^− 1^. *M_d_* is the actual torque in %, *M_d,unload_* is the idle torque in %, $\dot{m}$ is the total mass flow in kg h^− 1,^ and *P_max_* is the maximum engine power of 8600 W.

### 2.6. Sensory Evaluation of High-Moisture Texturized SPC Alpha 8

The HME samples were sensorial analyzed and clustered into well-textured, textured, and poorly-textured, respectively:

- Well-textured (=HMMA):

HME samples display firm and defined multilayered, fibrous structures, a pronounced parabolic pattern, and a well-defined V-shape pattern;

- Textured:

HME samples display a slightly fiber-like structure, a weak parabolic pattern when manually torn apart, as well as the trend of a V-shape pattern if cut longitudinally to the flow direction;

- Poorly-textured:

Soft, mushy, and brittle HME samples displaying no multilayered, fibrous structure, with a structure similar to shortcrust pastry.

### 2.7. Determination of Slice Shear Force of HME Samples Made out of SPC Alpha 8

**Figure 1:**
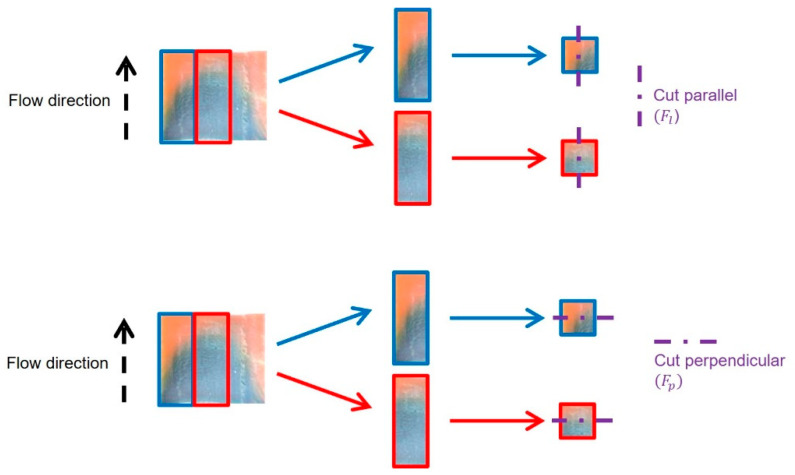
Schematic illustration of the sampling for the determination of slice shear force (SSF). Specimens were taken from the outer part and the middle of an HME sample.

The slice shear force (SFF) was determined according to the procedure of the American Meat Science Association [33] with small modifications. To determine differences in fiber-like structures, specimens were taken from the outer part as well as from the middle of an HME sample. Each specimen had a geometry of 20 × 20 × 9 mm^3^ (L × W × H). The sampling is shown in Figure 1, and the extruded samples were analyzed by cutting parallel ($F_{l}$) and perpendicular ($F_{t}$) to the direction outflow from the cooling die.

The specimens were cut using a flat, blunt-end blade with a thickness of 1 mm at a speed of 500 mm min^−1^. The cutting strength was recorded to determine the maximum SSF. All measurements were performed with at least five replicates.

The data from the SFF test were used to put the extruded samples in relation to their structure by creating a structuring index (*SI*). *SI* was determined by calculating the ratio $\frac{F_{l,m}}{F_{l,o}}$ with Equation (2), where *F_l,m_* is the shear slice force of the specimen taken from the middle of the HME sample, which was cut parallel, and *F_l,o_* is the shear slice force of the specimen taken from the outer part of the HME sample, which was cut parallel:

(2) $$SI\left[-\right]=\frac{F_{l,m}}{F_{l,o}}$$

### 2.8. Modeling of High-Moisture Texturization of SPC Alpha 8

The modeling approach focused on determining the mechanical and thermal energy input during high-moisture extrusion of SPC Alpha 8 by calculating the overall energy balance in the extruder using the first law of thermodynamics (Equation (3)):

(3) $$\Delta E_{Extruder}=\Delta U+\Delta E_{kin}+\Delta E_{pot}$$

where the internal energy ΔU consists of specific thermal energy (STE) and specific mechanical energy (SME) that have been supplied to the plant-based protein material (Equation (4)):

(4) ΔU=STE+SME

Chang et al. [34] considered, in their work with yellow corn meal, the material at a steady state as an incompressible flow system and defined the overall energy balance for an ideal adiabatic flow in the extruder. The approach by Chang et al. [34] assumes a negligible change in kinetic energy $\Delta E_{kin}$ when compared to the other terms and no potential energy change $\Delta E_{pot}$, since the extruder is horizontally oriented. Therefore, the specific energy input in the extruder can be calculated by using Equation (5):

(5) $$\Delta E_{Extruder}=STE+SME$$

If thermal loads for physical or chemical reactions can be neglected, the specific enthalpy of the plant-based protein material inside the extruder barrel could be described with Equation (6):

(6) $$\Delta H_{Extruder}=SME+STE=H_{0}-H_{in}=c_{p}\left(T_{in}-T_{0}\right)$$

where c_p_ is the specific heat capacity of the plant-based protein in J⋅K^−1^⋅kg^−1^. T_0_ is the initial material temperature in °C prior to extrusion, and T_in_ is the material temperature measured at the extruder exit, directly before the inlet of the cooling die in °C.

As mentioned before, physical or chemical reactions in the extruder barrel are not covered by Equation (6). However, a physical reaction that clearly occurs in the screw section is the phase transition. Phase transition, such as denaturation induced by protein melting, is an endothermic reaction, which can also be correlated to the melting temperature of the protein. A first-order phase transition such as protein denaturation is generally characterized by strong changes in enthalpy at the transition point [35,36,37]. To account for phase reaction-induced protein changes, the specific enthalpy of protein melting $\Delta H_{m}$ was added to the specific enthalpy of the extruder given above (Equation (5)):

(7) $$\Delta H_{Extruder}=STE+SME+\Delta H_{m}=c_{p}\left(T_{0}-T_{in}\right)+\Delta H_{m}$$

ΔH_m_ corresponded to the amount of heat required for converting the native state of the protein to a denatured state.

### 2.9. Implementation of a Texturization Indicator to Predict High-Moisture Texturization of SPC Alpha 8

A texturization indicator (TI) was implemented to model the high-moisture texturization of plant-based protein ingredients. TI is defined as the ratio of the specific extrusion enthalpy (ΔH_Extruder_) and the total enthalpy applied during DSC measurement (ΔH_DSC_) and is calculated by Equation (8):

(8) $$TI=\frac{\Delta H_{Extruder}}{\Delta H_{DSC}}=\frac{STE+SME+\Delta H_{m}}{\Delta H_{DSC}}$$

### 2.10. Validation to Predict High-Moisture Texturization of Plant-Based Proteins

The feasibility and applicability of the texturization indicator (TI) to predict high-moisture texturization was further tested for the plant-based proteins SPC Procon, SPI Wilcon, and PPI Pisane. Therefore, the methods depicted above for SPC Alpha 8 were also applied to investigate the behavior of SPC Procon, SPI Wilpro, and PPI Pisane during HME. However, for these plant-based protein materials, only a water content of 60% was studied. Table 2 shows the applied HME conditions to study the texturability of those plant-based proteins by using the same HME setup as described in detail for SPC Alpha 8.

### 2.11. Statistical Analysis

SigmaPlot 12.5 (Systat Software Inc., San Jose, CA, USA) was used for statistical analysis to evaluate significant differences between specific heat capacity values as well as in the structure of different textured HME samples by 1-way ANOVA with a probability of *p* < 0.05.

## 3. Results and Discussion

### 3.1. High-Moisture Texturization of SPC Alpha 8

The CCD results are listed in Table 3. For some of the extrusion settings, no SME could be recorded, and, therefore, some data records include a zero as a value for SME. A possible explanation for SME being zero respectively not measurable could be that the viscosity of the plant-based protein material in the extruder was too low due to high water content so that no measurable shear could be introduced through the screws [38,39]. Furthermore, several extrusion settings did not lead to protein texturization.

The sensory evaluation was linked to the HME process conditions to determine their impact on protein texturization. Extrusion conditions resulting in well-textured products (=HMMA products) were marked green, extrusion conditions that led to textured products (=threshold to HMMA status) were marked orange, and extruder conditions with a low degree of texturization (=poorly-textured samples) were marked red. As mentioned previously, for a few extruder settings, no protein texturization could be observed within the experimental CCD. As described in the Material and Methods section, the extruder settings were taken from the literature and were then implemented into the CCD and tested. Differences in extruder size and type, die configuration, cooling procedure, and/or raw materials might explain the difference in texturization [19,21]. These factors need to be considered for upscaling operations in the future [40].

**Figure 2:**
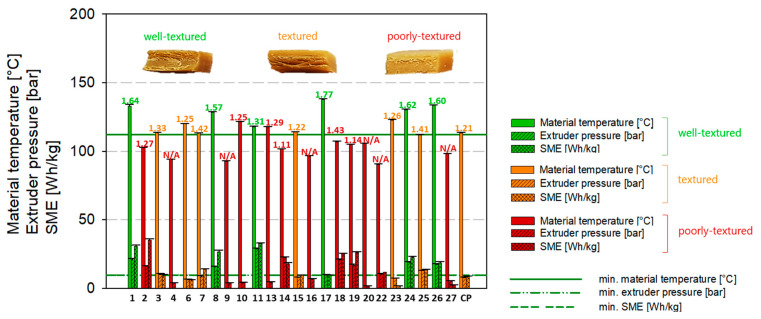
Texturization depending on extrusion conditions. The evaluation of well-textured, textured, and poorly-textured was carried out using subjective sensory qualities, including firmness, the presence of fiber-like structures, and the formation of a parabolic profile when torn apart. CP displays the mean value of the three central points (CPs) of the central composite design (CCD). Values on top of the column of each sample represent the calculated structuring index (Table 3).

However, for several extruder settings, HME samples, which were either well-textured or textured, were produced. The results of the extruder and product response are summarized and visualized in Figure 2.

Figure 2 shows the influence of extruder conditions, such as material temperature, extruder pressure, and SME, on the high-moisture texturization of SPC Alpha 8. The extruder responses of the three central points (CP; exp. 5, exp. 12, and exp. 21) within the experimental CCD were averaged over each output variable (Table 3). Therefore, mean material temperature, mean extruder pressure, and mean SME were calculated and plotted, which is the reason why 25 experiments instead of 27 experiments are displayed in Figure 2.

As depicted in Figure 2 on top of each column triplet, a structuring index (SI) was introduced as the degree of texturization, which serves as an indicator for the quality of structure formation [41]. The SI was implemented to get further insights into the influence of process conditions on texturization and was calculated by the force ratio *F_L,m_*/*F_L,o_* (Equation (2)) to display changes in product firmness caused by a variation of independent variables. It was chosen to introduce a new indicator for the quality of texturization instead of applying the anisotropy index (AI) commonly used in the literature to more accurately describe the flow profile, which is formed in the cooling die and, thus, the associated internal HMMA texture.

By comparing the quantitative texture analysis (=structuring index) with the qualitative texture analysis (=sensory evaluation in well-textured, textured, and poorly-textured) from Figure 2 and Table 3, the values of SI seem to correlate with the qualitative texture analysis. Statistical analyses showed that the SI of all well-textured samples (mean 1.54 ± 0.15) was significantly different (*p* < 0.05) from the textured (mean 1.26 ± 0.06) and poorly-textured HME samples (mean 1.25 ± 0.11). In contrast, no significant difference was found between the sensory-assessed textured and poorly-textured HME samples. Nevertheless, the combination of the quantitative and qualitative texture analysis has the potential to determine the degree o texturization of HME samples.

As shown in Figure 2, all HME products that exhibited multilayered, fiber-like structures, thus, all textured and well-textured defined samples, were extruded with material temperatures above 110 °C, revealing that the material temperature before entering the cooling die is a critical parameter for characteristics of HMES [41,42]. Well-textured samples (=HMMA) experienced even higher material temperatures in the screw section of the extruder (>117 °C) (Table 3 and Figure 2).

For the textured samples and the poorly-textured samples, it can be stated that either the material temperature >117 °C was not reached or the water content of the samples with 65–70% would have required higher energy input for a possible texturization. Further, for these trials, it should be noted that no or minor back pressure occurred in the cooling die, and the material compression in the cooling die might not be sufficient to form a pronounced laminar flow profile. The plant-based protein materials were cooled and solidified along the cooling die. However, no parabolic pattern was observed in the sample matrices, and no multilayered structure was formed.

As mentioned above, the SI was introduced to investigate in more detail the flow profiles formed in the cooling die as a function of the system responses, as the system parameters, e.g., material temperature and extruder pressure, have an impact on the texturization process in the cooling die, and, thus are successively responsible for texturization and structure formation [43,44].

**Figure 3:**
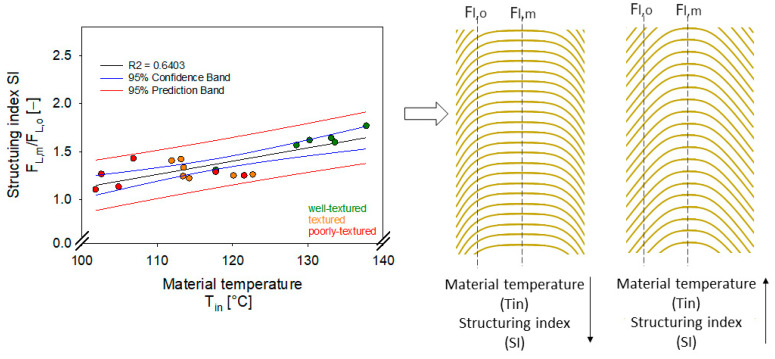
(**left**) Structuring index (SI) of HME samples as a function of material temperature. (**right**) Schematic illustration of the developed flow profile in the cooling die, which will be conserved in the HME sample due to solidification.

Therefore, SI was correlated to the different system parameters of the HME samples. The only parameter showing a linear correlation (R^2^ = 0.63) was the material temperature (Figure 3). In contrast, the extruder pressure and SME showed no linear correlation.

**Figure 4:**
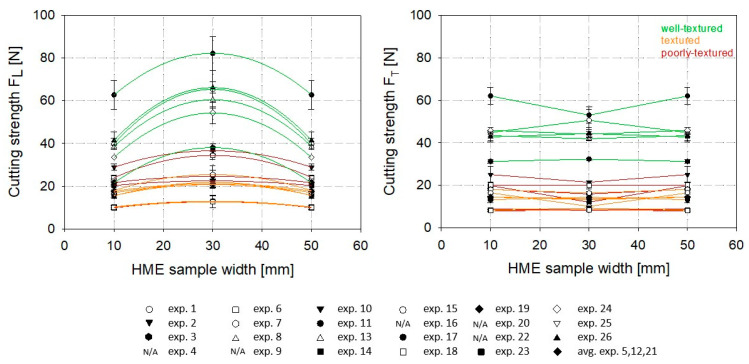
Cutting strength along the width of the extruded samples. Cutting strength was measured longitudinally (F_L_) (**left**) and transversely (F_T_) (**right**) to the flow in the cooling die. Samples were taken from the edge and center. Symmetry of high-moisture extruded samples was given, wherefore a mean cutting strength for the outer part was considered.

Nevertheless, the system parameters are interacting, and thus, extruder pressure and SME could have an impact on the structuring index, as well. This interaction could also be a reason for the relatively low determination coefficient (R^2^ = 0.64). Multiple linear regression modeling revealed no correlation between extruder pressure and SME but slightly better consistency for material temperature if linked to extruder pressure (R^2^ = 0.69) or SME (R^2^ = 0.74). Hence, extruder pressure and SME indirectly improved the structure formation. SME could promote the formation of a multilayered, fibrous structure by improving the mixing of the protein-water mixture [41,45]. The simplified linear description in Figure 3 reveals the relation between SI and material temperature since higher SI was measured at higher material temperatures. The schematic illustration in Figure 3 (right) highlights the alignment of fibrous structures in HME samples. The flow profile developed in the cooling die was more pronounced at higher material temperatures (Figure 4). This fact becomes clear by plotting the cutting strengths obtained at different positions (middle vs. center and longitudinally vs. transversally) within each HME sample (Figure 4). The longitudinally measured cutting forces increased towards the center. The geometry of the samples was assumed to be symmetrical; therefore, a mean cutting strength for both outer parts was considered. Based on the position, no significant differences in the cutting strengths measured transversally to the flow were discernable. The differences are mostly within the scope of the standard deviation (Figure 4).

To mathematically describe the changes in the longitudinally cutting strength F_L_ over the width of the HME samples (Figure 4 left), a quadratic curve fit was applied, which resulted in parabolic profiles, meaning that the firmness of the product is increasing towards the middle of the HME sample. This can be clearly shown for the HME experiments 01, 08, 11, 17, 24, and 26. These HME samples were defined as well-textured.

The pronounced flow profile of the well-textured HME samples might be due to several factors: the temperature gradient between the core product temperature and the cooling die wall is higher if the protein-water mixture enters the cooling die with a higher material temperature. This leads to a higher viscosity gradient, resulting in a steeper shear gradient and, thus, forming a more pronounced laminar flow profile with elongated longitudinal multilayered, fibrous structures.

Threshold temperatures for the formation of meat-like structures using HME have been discussed in the literature. Noguchi [42] observed a minimum HME temperature of 130 °C for soy flour at 60% moisture. Plant-based materials with high protein concentrations of 80–90% (e.g., soy and pea protein isolates) could be textured at different moisture contents (55–70%) and material temperatures below 130 °C [38,46,47].

Besides the importance of the threshold material temperature (>117 °C), the extruder pressure and SME need to be above the limit of 9 bar or 9 Wh/kg, respectively (Figure 2 and Table 3). Currently, there is limited data available for minimum values needed to be reached in the extruder for SME and extruder pressure, even though data exist supporting that extruder pressure increases firmness, toughness, and multilayered structures of HME products [38].

Based on the literature, the water content of the protein-water mixture has a significant influence on the degree of texturization [15,19,48]. This is in accordance with our HME experiments since an improved texturization with decreasing water content was demonstrated. Well-textured samples were primarily produced at water contents of 57.5% and 60%. HME samples with 65% moisture were mainly textured, and at 70% moisture, almost no texturization occurred. In the study of Lin et al. [46], samples extruded with a moisture content of 70% had a mushy structure, which was attributed to an incomplete texturization, and at higher moisture contents, e.g., 80% of water, no texturization occurred [41,46,49]. The increase in water content leads to the dilution of the initial protein concentration, which is why less protein is available to interact and form a protein melt [8,9].

Our results are in accordance with the results shown in the literature, although our study will introduce data-driven possibilities based on empirical knowledge to describe and anticipate the outcome of HME.

### 3.2. Determination of Specific Heat Capacity of SPC Alpha 8

The specific heat capacity for hydrated SPC Alpha 8 samples (60%, 65%, and 70% (*w*/*w* db)) was analyzed to calculate the energy transfer during extrusion and determine texturization properties. Small variations can be observed and might be attributed to minor variations in the natural raw material or in the sample’s moisture content incurred in either sample preparation or through relative humidity [50].

**Figure 5:**
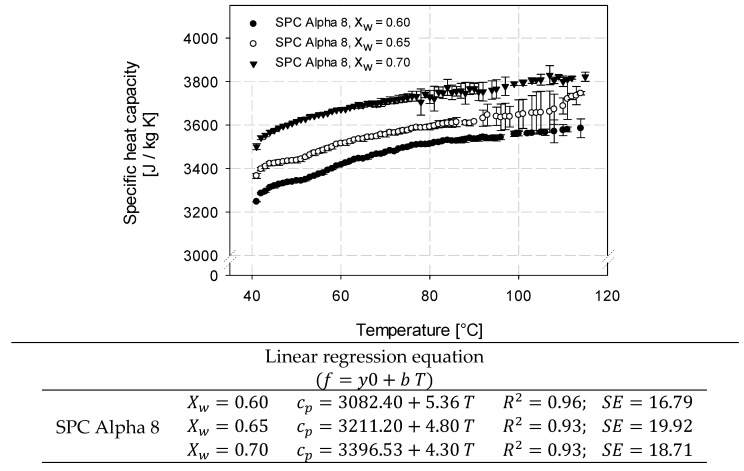
Specific heat capacity of soy protein concentrate (SPC Alpha 8) at water mass fraction (Xw) of 0.60, 0.65, and 0.70 (*w*/*w* db) as a function of temperature. The specific heat was determined using a microcalorimeter μDSC 7 EVO over the temperature range of 40–115 °C. For the linear model c_p_ is given in J/kg K and T in °C.

Wagner [50] used DSC measurements to measure the specific heat capacity for modeling the extrusion of soy flour. The studies revealed a denaturation reaction at an approximate temperature of 85 °C, but the occurrence of this reaction was concluded not to have any effect on the system, as the heat capacity of soy flour with moisture contents higher than 30% did not change. In our study, no effect of a denaturation reaction on the specific heat capacity could be seen for SPC Alpha 8 moistened up to 60%. Besides, such an influence was also not detected for HME samples made out of SPC Alpha 8 [31]. Thus, based on these preliminary runs, a denaturation effect on c_p_ could be eliminated. The results are shown in Figure 5 with c_p_ in J/kg K and T in °C.

**Figure 6:**
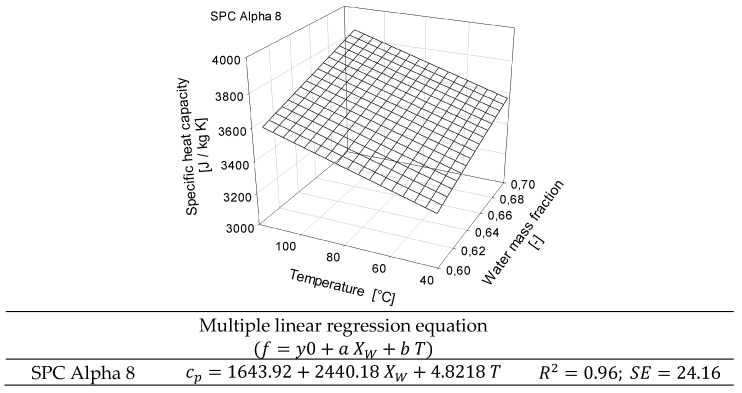
Effect of water mass fraction and temperature on the specific heat capacity of the plant-based protein concentrate SPC Alpha 8. The regression model is valid in the water content range of 60–70% (Xw = 0.6–0.7) and temperature range of 40–115 °C.

The specific heat capacity (c_p_) of all samples was linear with respect to temperature, and c_p_ increased with higher water content. This was to be expected since the higher the water content, the greater the energy that needs to be taken up by the system. The standard deviation ranged from 0.02 to 97.47, and higher deviations were found at higher temperatures. Linear regression equations of the SPC Alpha 8 samples at different moisture contents are listed in Figure 5. The results indicated significant differences (*p* < 0.05) for temperature and water mass fraction with respect to the specific heat capacity. Therefore, multiple linear regression (MLR) was performed to include the dominance of water, and the regression model is shown in Figure 6.

The predicted model showed a coefficient of determination (R^2^) of 0.96 and, thus, revealed a good correlation between experimental and predicted values. The specific heat capacity increased linearly with moisture content and temperature for all studied samples. The increase in heat capacity due to the addition of water was also to be expected, as the heat capacity of water is higher than that of protein [50]. In contrast to Wagner [50], an extrapolation of the data to 100% water showed higher values than the actual specific heat values of water. Therefore, our measured specific heat capacity could be slightly overestimated. The relative error of the extrapolated specific heat values estimating pure water increased with temperature. However, the relative error was below 10%, with a relative error of 2.2% at a temperature of 40 °C and 9.5% at 115 °C. The deviations for the experimentally determined c_p_ of distilled water compared to the literature showed a similar trend. The relative error also slightly increased with temperature, but all measurements were still in agreement with relative errors smaller than 10%. The relative errors in the measurements might be reduced by optimizing the µDSC method. Calorimetric measurements at lower heating rates (<0.2 K/min) could provide data that are more precise but would also extend the time needed for one measurement.

### 3.3. Determination of Phase Transition of SPC Alpha 8

**Figure 7:**
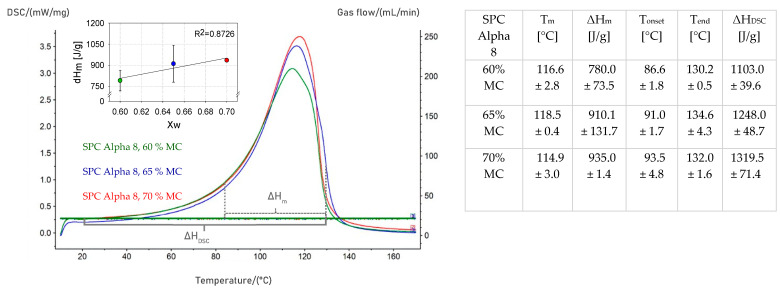
(**left**) Exemplary DSC thermograms of soy protein concentrate (SPC Alpha 8) at 60, 65, and 70% moisture (*w*/*w* db) heated at 5 K/min. (**right**) Results of phase transition.

The determination of phase transition was used to investigate texturization properties during HME and predict texturization conditions for HMMA with a defined multilayered, fibrous structure. By definition, the phase transition is the temperature at which the protein starts to denature [11,12]. This so-called melting temperature (T_m_) can be measured via DSC and, therefore, can be applied to conclude on texturability of protein ingredients during HME. Figure 7 displays an example DSC thermogram with the thermal behavior of each analyzed water content. The peak in the thermogram represents the thermally induced changes. Each thermogram was evaluated and characterized for the melting transitions of SPC Alpha 8 at different moisture contents by heat flow curves (dh/dt = f(T)) using the following calorimetric parameters: temperature of initial deviation of heat flow (T_onset_), the maximum temperature of peak (T_m_), and end temperature (T_end_) [10]. The integration of heat flow to temperature in the temperature range T_onset_ to T_end_ yields a value for the enthalpy change (ΔH_m_). By integrating the heat flow over the temperature from T_0_ to T_end_, the enthalpy needed to develop a complete protein melt can be calculated. In further discussion, this will be referred to as enthalpy applied during DSC measurement (ΔH_DSC_). The phase transition was evaluated over the temperature range of 20 °C to 170 °C; thus, the material temperatures measured in the HME experiments were included in the DSC measurements. Preliminary tests were performed using a µDSC, which, compared to DSC, is a more sensitive device with low adjustable heating rates and improved resolution for recording even small changes in the heat flux [51]. The resulting thermogram was similar to the thermogram demonstrated in Figure 7. However, two peaks were depicted in the µDSC scan, showing a very small peak at a temperature of approximately 80 °C (Δ = 0.5 J/g) and a second peak starting at approximately 95 °C and rising above the device’s temperature maximum (<120 °C). Due to these results, the measurements of the phase transition for the hydrated SPC Alpha 8 samples (60%, 65%, and 70%) were carried out using a standard DSC device since it can cover a broader measuring temperature range.

The DSC thermograms presented in Figure 7 are examples of repetitive measurements. Small variations between the multiple measurements could be noticed, which might be due to natural variations of the raw material, relative humidity, or moisture content of the sample due to preparation. For all three analyzed moisture contents, the obtained DSC thermograms revealed one broad peak. No significant differences were found between the onset, midpoint, and end temperatures for the different water contents (*p* > 0.05). The denaturation temperature of the globulins in lupin protein isolates was found to be a function of moisture content (50–70%) [52]. Differences in the specific melting enthalpy for various moisture content could be seen, even though those differences were not significant (*p* > 0.05). The specific enthalpy increased with increasing water. Wagner [50] also found no correlation between the energy associated with the endothermic reaction and the moisture content in soybean flour.

According to the literature, the endothermic peak in the DSC thermograms is representative of the denaturation of the main soy fractions, β-conglycinin (7S), and glycinin (11S), which denature at about 85 °C (7S) and 107 °C (11S), respectively [50,52,53]. The endotherm of β-conglycinin (7S) is very small and might be too small to be noticed in DSC measurements or is incorporated in the revealed peak. As mentioned, it could be detected in the preliminary test performed using µDSC. The obtained DSC results were similar to the findings in the literature, although it was partially reported that the majority of commercial soy protein products showed no DSC endotherms [52]. Sousa et al. [52] found temperature peaks at 91 °C (7S) and at 111 °C (11S) for SPI at 50% moisture. For SPI at 64% water content, midpoint temperatures at 102.3 °C (7S) and 124 °C (11S) were detected [49]. For soy flour at 50% moisture, one main peak at approximately 115 °C was obtained in the studies of Wagner [50]. Wagner [50] studied enthalpy changes in soy flour at different water contents (0–70%), but thermograms without exact data were reported. Differences in the denaturation temperature can be ascribed to differences in protein origin and composition as well as to analysis performance since phase transition is also influenced by the heating rate [54].

### 3.4. Modeling of High-Moisture Texturization of SPC Alpha 8

The results of the DSC study and the calculated energy input in the extruder barrel for the HME trials were correlated to estimate the amount of mechanical and thermal energy needed for texturization during HME.

**Figure 8:**
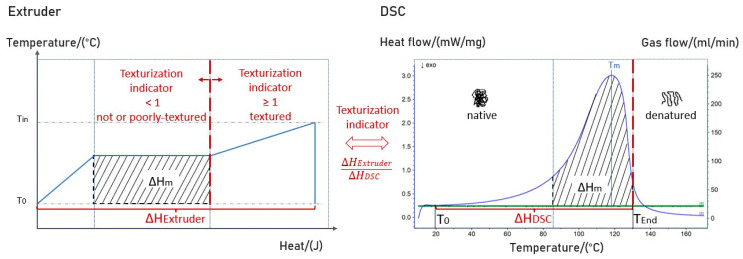
Schematic illustration of extrusion enthalpy in comparison to enthalpy determined using DSC measurements. A texturization indicator was introduced that describes the ratio between extrusion enthalpy and enthalpy needed for the complete formation of a protein melt obtained by DSC measurements.

As mentioned previously, no or fewer fibrous structures after HME were attributed to a possible incomplete texturization and insufficient protein denaturation during protein melting [42,46,49]. Therefore, we postulate that a complete protein denaturation leading to texturization during HME would occur at an extrusion enthalpy greater than the enthalpy delivered to the plant-based protein using DSC, including the enthalpy for heating until protein melting (Figure 8). The formation of protein melting, which starts at T_m_ and leads to protein denaturation, can be considered the initial and most important step for texturization [13,14].

To validate and test our hypothesis, a texturization indicator (TI) was introduced that determined the relation of extrusion enthalpy to the enthalpy needed to form a complete protein melt obtained by DSC measurements.

**Figure 9:**
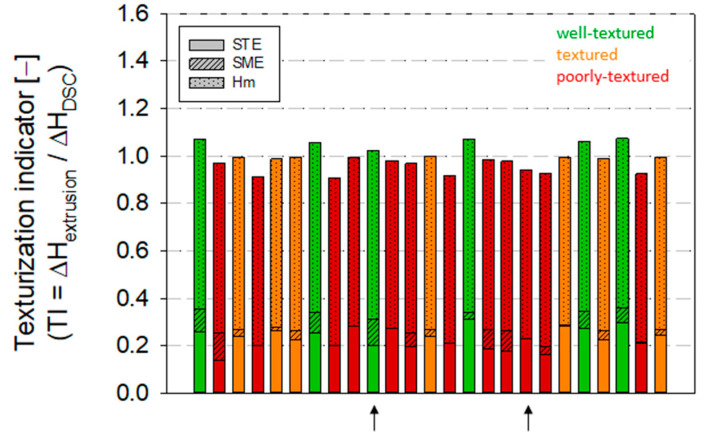
Texturization indicator (TI), as the ratio of extrusion enthalpy to DSC enthalpy for a complete protein melt. Well-textured and textured HME samples having a TI ≥ 1. HME samples that were poorly-textured have a TI < 1. The classification of well-textured, textured, and poorly-textured was carried out by sensory evaluation. The arrow indicates experiments for which the DSC enthalpies had been extrapolated.

For each HME experiment (Table 3), the extrusion enthalpy (∆H_Extruder_) was determined based on Equation (7), and TI was calculated according to Equation (8) with the enthalpy needed for the complete formation of a protein melt (∆H_DSC_) from Figure 7. For the specific heat capacity, the multiple linear regression (MLR) equation for hydrated SPC Alpha 8 derived in this study was applied, and the mean temperature over the entire temperature range (T_0_ to T_in_) in the extruder barrel was considered for its calculation. An initial temperature (T_0_) of 20 °C was designated. The enthalpies ΔH_m_ for different water contents listed in Figure 7 were used as melting enthalpies. The total enthalpies applied during DSC measurement ΔH_DSC_ were calculated in the temperature range of T_0_ to T_End_ (Figure 7). The calculated texturization indicator was further correlated with the sensory evaluation of the high-moisture extruded samples (Table 3). The results are shown in Figure 9.

TI > 1 would assume the formation of a fully developed protein melt in the screw section of an extruder. On the other hand, TI < 1 would result in no formed or a partially formed protein melt, leading to an incomplete texturization.

To obtain an outcome that would meet the definition of a well-textured HME sample, our data (Figure 9) suggests that TI should be above 1, ideally between 1.05–1.1.

For two HME experiments, which were extruded with a water content of 57.5 and 72.5%, respectively, the related melting enthalpy and total DSC enthalpy were extrapolated from the DSC data. These HME experiments are marked with an arrow in Figure 9. As shown in Figure 9, a TI ≥ 1 could be calculated for the textured or well-textured HME samples. Poorly-textured products had a TI < 1, evidencing that partial or no denaturation of the protein mass occurred in those HME experiments; the energy supplied in the extruder barrel was not sufficient to form a complete protein melt. The well-textured HME sample had an average TI of 1.07 ± 0.01, the textured products had an average TI of 1.00 ± 0.01, and products with poor or without texture had an average value of 0.95 ± 0.03. The calculated data confirms the sensory evaluation, revealing that the introduced texturization indicator can reflect the texture of HME samples and, thus, can be used to model the texturability of plant-based proteins and to optimize the high-moisture texturization to produce HMMA products with defined fibrous structures. Therefore, to predict the texturability of plant-based protein materials prior to extrusion, the minimum material temperature (T_in_) required to form a complete protein melt during HME could be calculated by rearranging Equation (9):

(9) $$TI=\frac{\Delta H_{Extruder}}{\Delta H_{DSC}}=\frac{STE+SME+\Delta H_{m}}{\Delta H_{DSC}}$$

(10) $${TI\times \Delta H}_{DSC}={\Delta H}_{Extruder}$$

(11) $${TI\times \Delta H}_{DSC}=c_{p}\left(T_{in}-T_{0}\right)+{\Delta H}_{m}$$

(12) $$T_{in}=\frac{TI\times {\Delta H}_{DSC}-{\Delta H}_{m}}{c_{p}}+T_{0}$$

with TI≥1 for textured or well-textured HME samples

(13) $$T_{in}\leq \frac{{\Delta H}_{DSC}-{\Delta H}_{m}}{c_{p}}+T_{0}$$

The minimum texturization temperatures for SPC Alpha 8 at different water contents were calculated according to Equation (12), and the multiple linear regression for the specific heat capacity of SPC Alpha 8 derived in this study was applied. However, in contrast to the previous calculation, the initial temperature (T_0_ = 20 °C) was used within this regression equation since the material temperature T_in_ was unknown in this case. Therefore, the calculated texturization temperatures listed in Table 4 might be slightly overestimated.

A linear dependence between calculated texturization temperature and water content was found, and in our study, each well-textured HME sample was extruded with a material temperature higher than its calculated texturization temperature. For the textured samples, the material temperature was equal to or slightly higher than the calculated temperature, and the material temperature for the poorly-textured HME samples was predominantly below the calculated temperature (Table 3 and Figure 2). In comparison to the quantitative (=structuring index) and qualitative (=sensory evaluation) texture methods discussed in Figure 2, the use of TI seems to be more precise, and more importantly, it can be carried out prior to HME processing.

### 3.5. Validation to Predict High-Moisture Texturization of Plant-Based Proteins

The approach used to predict the texturability for SPC Alpha 8 by calculating the minimum texturization temperatures (T_in_) based on DSC measurements (Table 4) in combination with the introduced TI (Figure 9) was tested for other plant-based proteins, such as SPC Procon, SPI Wilcon, and PPI Pisane. Besides SPC Alpha 8, these plant-based protein materials are used in industry as raw materials for HME. The DSC results of c_p_ and phase transition are shown in Table 5.

By using Equation (12) with these DSC data, the minimum texturization temperatures (T_in_) for the different plant-based proteins at a water content of 60% were calculated. The minimum texturization temperature (T_in_) could be understood as the extrusion melting hurdle (EMH) that has to be crossed in order to generate a complete protein melt in the screw section and, thus, to produce HMMA products with a defined texture. The calculated minimum texturization temperatures (T_in_) are summarized in Table 6.

In addition, HME tests were carried out with the different plant-based proteins to validate the calculated minimum texturization temperatures (Table 6). Different barrel temperatures were intentionally selected to specifically generate different HMES textures (poor-textured, textured, well-textured). The HME result of each HME experiment was compared to the respective minimum texturization temperature (T_in_), which was calculated using Equation (12). A mean temperature based on the feed temperature (T_0_ = 20 °C) and the material temperature (T_in_) was used for the calculation of c_p_ of the different plant-based proteins (Table 5). Table 7 shows the applied process conditions and the process responses in comparison to the calculated minimum texturization temperature and TI.

Table 7 summarizes the results of the validation study to predict high-moisture texturization of the plant-based proteins SPC Procon, SPI Wilpro, and PPI Pisane. As already shown for SPC Alpha 8 (Table 3 and Figure 9), well-textured HME samples could be extruded with material temperatures higher than the calculated texturization temperature. For textured HME samples, the material temperature was equal to the calculated texturization temperature, and the material temperature of the poorly-textured sample was lower than the minimum calculated temperature. Furthermore, this study clearly showed that all the plant-based protein materials studied had different minimum texturization temperatures, and the calculated texturization temperatures of the plant-based proteins SPC Procon, SPI Wilcon, and PPI Pisane (Table 7) were higher compared to that of SPC Alpha 8 (Table 4) at the same water content (Xw = 0.6, *w*/*w* db). The calculated data confirm the sensory evaluation (Table 7) and demonstrate that the introduced TI can be applied to determine the minimum temperature required for protein texturization and, therefore, can be used to model the texturability of different plant-based proteins prior to HME trials.

## 4. Conclusions

The results show that calorimetric methods can be used to estimate process conditions based on thermophysical properties prior to HME. In our study, the texturization indicator (TI) to predict the texturability of plant-based proteins by introducing the extrusion melting hurdle (EMH) temperature as the minimum temperature for protein texturization was developed. EMH represents the temperature that needs to be surpassed in the extruder barrel to induce sufficient protein melting and structure formation to generate HMMA.

Therefore, based on the results obtained in our study, it can be concluded that the EMH temperature to reach the minimum texturization degree differs between plant-based proteins at 60% water content (*w*/*w* db). Thus, SPC Alpha 8 requires a minimum texturization temperature of 117.7 °C, SPC Procon requires a minimum temperature of 128.0 °C, SPI Wilpro requires a minimum temperature of 124.2 °C, and PPI Pisane requires a minimum temperature of 120.4 °C. Consequently, the calorimetric approach should be handled as a case-by-case approach since specific heat capacity and phase transition parameters need to be assessed for each protein prior to HME for HMMA development.

Nevertheless, the approach represents a valid tool for finding optimal texturization conditions, and it offers advantages, such as convenient preparation, rapid analysis, and a high potential to minimize resources and rationalize product development without trial and error experimentation. Furthermore, it could improve ingredient flexibility for HME processing and could be used to predict the texturability of protein mixtures or proteinaceous flours.

## Figures and Tables

**Table 1:** Central composite design experiments. X1: Screw speed [rpm]; X2: Moisture content [%]; X3: Mass flow rate [kg/h]; X4: Barrel temperature [°C].

Experiment Number	Experimental Plan			
	X_1_	X_2_	X_3_	X_4_
1	400	60	13	160
2	400	60	6	120
3	175	65	9.5	140
4	220	70	6	120
5	310	65	9.5	140
6	400	70	13	160
7	445	65	9.5	140
8	400	60	6	160
9	400	70	6	120
10	220	70	6	160
11	310	57.5	9.5	140
12	310	65	9.5	140
13	400	70	6	160
14	220	60	13	120
15	310	65	4.25	140
16	220	70	13	120
17	310	65	9.5	170
18	220	60	6	120
19	400	60	13	120
20	310	72.5	9.5	140
21	310	65	9.5	140
22	310	65	9.5	110
23	220	70	13	160
24	220	60	13	160
25	310	65	14.25	140
26	220	60	6	160
27	400	70	13	120

**Table 2:** Design of experiments for the validation study with the plant-based proteins SPC Procon, SPI Wilpro, and PPI Pisane: X1: Screw speed [rpm]; X2: Moisture content [%]; X3: Mass flow rate [kg/h]; X4: Barrel temperature [°C].

Experiment Number	Experimental Plan			
	X_1_	X_2_	X_3_	X_4_
SPC Procon No.1	400	60	13	130
SPC Procon No.2	400	60	13	140
SPI Wilpro No.1	400	60	13	120
SPI Wilpro No.2	400	60	13	130
PPI Pisane No.1	400	60	9	130
PPI Pisane No.2	400	60	9	155

**Table 3:** Central composite design experiments and the corresponding experimental responses.

Experiment Number	Experimental Plan				Responses				
					Process Response			Product Response	
	X_1_	X_2_	X_3_	X_4_	Y_1_	Y_2_	Y_3_	Texture	
								Quantitative Structuring index	Qualitative Sensory
1	400	60	13	160	133.10	21.06	30.68	1.64	well-textured
2	400	60	6	120	102.57	16.20	35.34	1.27	poorly-textured
3	175	65	9.5	140	113.49	10.54	9.28	1.33	textured
4	220	70	6	120	93.90	3.84	0	N/A	poorly-textured
5 (CP)	310	65	9.5	140	116.84	7.96	9.78	1.14	textured
6	400	70	13	160	120.11	6.63	6.09	1.25	textured
7	445	65	9.5	140	113.15	8.40	13.98	1.42	textured
8	400	60	6	160	128.48	15.95	26.74	1.57	well-textured
9	400	70	6	120	92.62	3.80	0	N/A	poorly-textured
10	220	70	6	160	121.53	4.03	0	1.25	poorly-textured
11	310	57.5	9.5	140	117.76	28.80	32.75	1.31	well-textured
12 (CP)	310	65	9.5	140	114.44	8.94	8.62	1.23	textured
13	400	70	6	160	117.73	4.47	0	1.29	poorly-textured
14	220	60	13	120	101.80	22.47	17.86	1.11	poorly-textured
15	310	65	4.25	140	114.22	7.98	9.81	1.22	textured
16	220	70	13	120	96.28	6.84	0	N/A	poorly-textured
17	310	65	9.5	170	137.76	9.69	9.71	1.77	well-textured
18	220	60	6	120	106.82	20.96	25.34	1.43	poorly-textured
19	400	60	13	120	104.85	16.73	26.20	1.14	poorly-textured
20	310	72.5	9.5	140	105.67	1.61	0	N/A	poorly-textured
21 (CP)	310	65	9.5	140	110.95	6.67	6.72	1.26	textured
22	310	65	9.5	110	90.42	10.49	11.85	N/A	poorly-textured
23	220	70	13	160	122.68	7.51	1.73	1.26	textured
24	220	60	13	160	130.22	19.08	22.70	1.62	well-textured
25	310	65	14.25	140	111.89	12.91	13.51	1.41	textured
26	220	60	6	160	133.58	17.45	18.70	1.60	well-textured
27	400	70	13	120	98.18	5.44	2.38	N/A	poorly-textured

X_1_: Screw speed [rpm]; X_2_: Moisture content [%]; X_3_: Mass flow rate [kg/h]; X_4_: Barrel temperature [°C]. Y_1_: Material temperature [°C]; Y_2_: Extruder pressure [bar]; Y_3_: SME [Wh/kg]; N/A.: not applicable. CP: central point.

**Table 4:** Minimum texturization temperatures for high-moisture texturization of SPC Alpha 8 at different water contents. The calculation was based on Equation (10).

Water Content	[%]	57.5	60	65	70	72.5
Minimum texturization temperature	[°C]	112.70	117.68	121.47	131.50	133.59

**Table 5:** Thermophysical properties of plant-based proteins SPC Procon, SPI Wilpro, and PPI Pisane. (left) Effect of water mass fraction and temperature on specific heat capacity. The regression models are valid in the water content range of 60–70% (Xw = 0.6–0.7, *w*/*w* db) and temperature range of 40–115 °C. (right). Results of phase transition at water content range of 60% (Xw = 0.6).

Specific Heat Capacity c_p_			Phase Transition at Xw = 0.6				
Multiple linear regression equation $\left(f=y0+aX_{W}+bT\right)$			T_m_ [°C]	ΔH_m_ [J/g]	T_onset_ [°C]	T_end_ [°C]	ΔH_DSC_ [J/g]
SPC Procon	$c_{p}=1675.38+2408.56X_{W}+4.6196T$	$R^{2}=0.96$	115.9	834.1	86.0	129.9	1205
		SE=24.16	±4.7	±13.5	±3.4	±3.5	±29.6
SPI Wilpro	$c_{p}=1867.48+2393.64X_{W}+2.6710T$	$R^{2}=0.92$	114.6	673.2	83.4	126.5	1036
		SE=29.41	±0.4	±31.7	±1.7	±2.3	±8.7
PPI Pisane	$c_{p}=1939.57+1939.20X_{W}+4.6319T$	$R^{2}=0.94$	117.9	781.4	90.0	133.6	1125
		SE=26.41	±0.2	±1.2	±2.8	±0.6	±41.4

**Table 6:** Minimum texturization temperatures (T_in_) for different plant-based proteins at a moisture content of 60% (*w*/*w* db). T_in_ was calculated based on Equation (12).

Protein	Moisture Content	Calculated Minimum Texturization Temperature T_in, calculated_
SPC Procon	60%	128.00 °C
SPI Wilpro	60%	124.15 °C
PPI Pisane	60%	120.40 °C

**Table 7:** Results of the validation study to predict high-moisture texturization of plant-based proteins SPC Procon, SPI Wilpro, and EPI Pisane. The results include the design of experiments, the experimental responses, the calculated minimum texturization temperature, as well as the texturization indicator.

Protein	Experiment No.	Experimental Plan				Extrusion Response			Calculated min. Texturization Temperature
		X_1_	X_2_	X_3_	X_4_	Pressure [bar]	SME [Wh/kg]	T_in_ [°C]	T_in_ [°C]
SPC Procon	No.1	400	60	13	130	40.00	42.47	125.17	128.00
	No.2	400	60	13	140	38.50	40.00	137.20	128.00
SPI Wilpro	No.1	400	60	13	120	30.18	33.72	109.59	124.15
	No.2	400	60	13	130	41.10	48.90	129.70	124.15
PPI Pisane	No.1	400	60	9 †	130	16.60	16.20	120.10	120.40
	No.2	400	60	9 †	155	12.40	11.77	134.15	120.40
**Protein**	**Experiment No.**	**Product** **response**							**Texturization** **indicator** based on Equation (8)
		Qualitative Sensory			Product Images				TI ‡ [−]
SPC Procon	No.1	poorly-textured			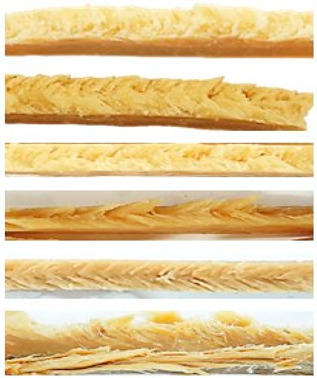				0.99
	No.2	well-textured							1.03
SPI Wilpro	No.1	poorly-textured							0.95
	No.2	well-textured							1.02
PPI Pisane	No.1	textured							1.00
	No.2	well-textured							1.05

X_1_: Screw speed [rpm]; X_2_: Moisture content [%]; X_3_: Mass flow rate [kg/h]; X_4_: Barrel temperature [°C]. † The mass flow rate for PPI Pisane was 9 instead of 13 kg/h, but since the comparison of texturization was only conducted for the same raw material, this fact could be neglected. Further, this could also show that this approach might be independently applicable in a certain mass flow rate domain. ‡ TI > 1: well-textured; TI = 1: textured, and TI < 1: poorly-textured.

## Data Availability

The data presented in this study are available on request from the corresponding author.
